# Electrophysiological Evidence for a Direct Link between the Main and Accessory Olfactory Bulbs in the Adult Rat

**DOI:** 10.3389/fnins.2015.00518

**Published:** 2016-01-26

**Authors:** Victor Vargas-Barroso, Benito Ordaz-Sánchez, Fernando Peña-Ortega, Jorge A. Larriva-Sahd

**Affiliations:** Neurobiología del Desarrollo y Neurofisiología, Instituto de Neurobiología, Universidad Nacional Autónoma de México, Campus JuriquillaQuerétaro, México

**Keywords:** accessory olfactory bulb, main olfactory bulb, bursting, patch-clamp

## Abstract

It is accepted that the main- and accessory- olfactory systems exhibit overlapping responses to pheromones and odorants. We performed whole-cell patch-clamp recordings in adult rat olfactory bulb slices to define a possible interaction between the first central relay of these systems: the accessory olfactory bulb (AOB) and the main olfactory bulb (MOB). This was tested by applying electrical field stimulation in the dorsal part of the MOB while recording large principal cells (LPCs) of the anterior AOB (aAOB). Additional recordings of LPCs were performed at either side of the plane of intersection between the aAOB and posterior-AOB (pAOB) halves, or *linea alba*, while applying field stimulation to the opposite half. A total of 92 recorded neurons were filled during whole-cell recordings with biocytin and studied at the light microscope. Neurons located in the aAOB (*n* = 6, 8%) send axon collaterals to the MOB since they were antidromically activated in the presence of glutamate receptor antagonists (APV and CNQX). Recorded LPCs evoked orthodromic excitatory post-synaptic responses (*n* = 6, aAOB; *n* = 1, pAOB) or antidromic action potentials (*n* = 8, aAOB; *n* = 7, pAOB) when applying field stimulation to the opposite half of the recording site (e.g., recording in aAOB; stimulating in pAOB, and vice-versa). Observation of the filled neurons revealed that indeed, LPCs send axon branches that cross the *linea alba* to resolve in the internal cellular layer. Additionally, LPCs of the aAOB send axon collaterals to dorsal-MOB territory. Notably, while performing AOB recordings we found a sub-population of neurons (24% of the total) that exhibited voltage-dependent bursts of action potentials. Our findings support the existence of: 1. a direct projection from aAOB LPCs to dorsal-MOB, 2. physiologically active synapses linking aAOB and pAOB, and 3. pacemaker-like neurons in both AOB halves. This work was presented in the form of an Abstract on SfN 2014 (719.14/EE17).

## Introduction

Successful decoding of complex environmental stimuli by the nervous system relies upon central convergence of primary sensory systems. An important example of this is the interaction between the main- (MOS) and accessory olfactory (AOS) systems (Suárez et al., 2012; Baum and Larriva-Sahd, 2014). In fact, volatile and pheromonal stimuli that are sensed by the MOS and AOS, respectively, bring about functionally and behaviorally overlapping responses in these systems (Sam et al., 2001; Trinh and Storm, 2003; Lin et al., 2004; Xu et al., 2005; Spehr et al., 2006; Larriva-Sahd, 2008, 2012b). In the absence of a structural interaction between the main olfactory epithelium (MOE) and the vomeronasal organ (VNO) or between the main- (MOB) and accessory olfactory (AOB) bulbs, synergistic responses of the MOS and AOB are largely attributed to the anatomical overlap beyond these primary and secondary sensory structures, respectively (Boehm et al., 2005; Kang et al., 2011).

At the cell receptor level, volatile stimuli bind distinct sets of olfactory receptors in the MOE (Buck and Axel, 1991; Mori et al., 1999) while pheromonal cues are actively pumped into the VNO (Mann, 1961; Meredith and O'Connell, 1979), where sensory cells are activated by the later (Leinders-Zufall et al., 2000; Boschat et al., 2002; Del Punta et al., 2002). Furthermore, sensory cells in the MOE and VNO project their central processes to the MOB (Ramón y Cajal, 1890) and AOB (Barber and Raisman, 1974; Larriva-Sahd, 2008), respectively. Mitral neurons in the MOB project to secondary olfactory areas (Ojima et al., 1986; Stettler and Axel, 2009; Kang et al., 2011), whereas in the AOB, large principal cells (LPCs; Larriva-Sahd, 2008) project to the so-called vomeronasal amygdala (Scalia and Winans, 1975; Boehm et al., 2005; Mohedano-Moriano et al., 2007; Kang et al., 2011).

It has been shown that the AOB is directly implicated in decoding pheromonal stimuli (Leinders-Zufall et al., 2000; Boschat et al., 2002; Del Punta et al., 2002; Luo et al., 2003) and that these chemical cues are detected by two sub-systems within the AOB (Imamura et al., 1985; Mori et al., 1987). For instance, VNO sensory cells distribute in two layers, apical and basal (Dulac and Axel, 1995) in which G protein expression (Berghard and Buck, 1996) and projection targets toward the AOB (Mori et al., 1987) vary as a function of location. More specifically, apical cells that express vomeronasal receptor 1 (V1R) family of receptors (Dulac and Axel, 1995) and most members of the formyl-peptide receptor (FPR) family present in the VNO (Liberles et al., 2009; Riviere et al., 2009), project their axons to the anterior half of the AOB (aAOB), whereas basal cells that express members of the vomeronasal 2 receptor (V2R) family (Herrada and Dulac, 1997; Matsunami and Buck, 1997; Ryba and Tirindelli, 1997) project to the posterior half of the AOB (pAOB; Mori et al., 1987; Schwarting et al., 1994). Further, apical cells synapsing in the aAOB bind pheromones present in the urine of conspecifics (Leinders-Zufall et al., 2000; Boschat et al., 2002; Del Punta et al., 2002), some of which are sulfated steroids (Nodari et al., 2008; Hammen et al., 2014), whereas basal cells that synapse in the pAOB bind high molecular weight molecules such as major urinary proteins (MUPS; Chamero et al., 2007), as well as peptide ligands of major histocompatibility complex (MHC) proteins (Leinders-Zufall et al., 2004) and peptides derived from extraorbital lacrimal glands (Kimoto et al., 2005). Thus, these relatively independent streams of information processing within the AOS deal with specific semiochemicals and may have different implications for behavior and survival. It has even been shown that their projections into the brain are also partially segregated (Mohedano-Moriano et al., 2007), and as with a putative interaction between the MOB and the AOB, direct crosstalk between the two AOB halves remains to be demonstrated.

Fundamental for the present study is that anatomical overlap between the MOS and AOS has only been documented in secondary and tertiary projections arising from the MOB and AOB to the basal forebrain (Boehm et al., 2005; Kang et al., 2011; Mohedano-Moriano et al., 2012) The parceled, yet parallel, central path of vomeronasal, and main olfactory projections led to the dualistic notion that the MOS and AOS constitute distinct sensory systems (Raisman, 1972; Scalia and Winans, 1975). More recently, a growing number of observations which in most respects, confirmed earlier ones, have depicted varying degrees of structural overlap in both secondary relays of the MOS (nucleus of the lateral accessory tract, anterior cortical nucleus of the amygdala, and piriform-amygdaloid transitional zone of the amygdala; Shammah-Lagnado and Negrao, 1981; Dong et al., 2001) and AOS (ventral anterior, bed nucleus of the accessory olfactory tract, and medial amygdaloid nucleus; see Hintiryan et al., 2012). Furthermore, third order olfactory and vomeronasal recipient areas converge in the basal forebrain (see Dong et al., 2001; Dong and Swanson, 2004). In contrast, tract tracing studies aimed at defining a possible direct connection between the mammalian MOB and AOB have been unsuccessful (Price, 1973; Hintiryan et al., 2012, but see Martínez-García et al., 1991).

Thus, although these data may be the starting point for a new integrated hypothesis concerning chemosensory detection and processing, there has not been any information regarding the anatomical substrate of interactions at the level of the olfactory bulbs (but see Martínez-García et al., 1991). With the aim of obtaining physiological evidence for interactions between the MOB and the AOB and between the two AOB halves, we performed whole-cell patch-clamp recordings of neurons in the aAOB and pAOB while applying electrical stimuli to the MOB and/or to the AOB opposite to the recording site. Given the numerous connectional and cytological differences between the MOB and the AOB mitral cells, we have adopted the term LPC to refer to the “mitral cell” of the former (see Larriva-Sahd, 2008). Our results provide evidence that a subset of aAOB LPCs project to the dorsal-posterior MOB and that both AOB halves are synaptically connected. In addition, single cell recordings disclosed that, like in the MOB (Hayar et al., 2004a,b; Liu and Shipley, 2008), the AOB harbors a population of neurons with voltage-dependent intrinsic bursting activity.

## Materials and methods

### Animals

For this study, male Wistar rats of 8 weeks of age were used for whole-cell recordings. Animal manipulation and sacrifice were performed under the guidelines and with the approval of the Animal Research Committee of our Institute, which endeavors to minimize pain and suffering to the experimental subjects.

### Olfactory bulb slice preparation

Rats were anesthetized with pentobarbital (63 mg/Kg) and perfused intracardially with an ice-cold modified artificial cerebrospinal fluid (aCSF) that contained (in mM): 238 sucrose, 3 KCl, 2.5 MgCl_2_, 25 NaHCO_3_, and 30 D-glucose (pH 7.4; Alvarado-Martínez et al., 2013). The bulbs were removed and placed in normal aCSF equilibrated with carbogen (95% O_2_ and 5% CO_2_). The aCSF contained (in mM): 119 NaCl, 3 KCl, 1.5 CaCl_2_, 1 MgCl_2_, 25 NaHCO_3_, and 30 D-glucose (pH 7.4). The medial end of either the right or the left bulb was glued to a block of agar, mounted on a vibratome (Leica VT1000S), and serially cut into 350-μm thick slices obtained on a sagittal plane. Two or three slices per bulb were allowed to recover for 1 h in aCSF bubbled with carbogen at room temperature. Finally, a single slice was transferred to a recording chamber (3 ml) located on a Nomarski-DIC-equipped microscope (Eclipse E600FN; Nikon, Melville, NY). The chamber was continuously superfused with oxygenated aCSF (32 ± 0.5°C) at a flow rate of 17 ml/min. A total volume of 30 ml of recirculating medium was maintained both in the chamber and the tubing system.

### Electrophysiological recordings

Whole-cell patch-clamp recordings were obtained using the visual patch-clamp technique with an Axo-clamp 2B amplifier (Axon Instruments, Foster City, CA; Peña et al., 2010). Cells were aimed for and recorded based on their location within the external cellular layer of the AOB; those situated in the aAOB (*n* = 72) constitute the bulk of the recordings, and a small sample of pAOB (*n* = 20) neurons were also recorded. Patch electrodes (4–8 MΩ) were pulled from filamented borosilicate glass tubes (G150F-4; Warner Instruments, Handem, CT) and filled with a solution containing (in mM): 140 K-gluconic acid, 10 EGTA, 2 MgCl_2_, 10 HEPES, 2 of Na_2_ATP, 2 of LiGTP, and 1% biocytin, pH 7.4. Recordings performed with either lithium (i.e., LiGTP) or magnesium (i.e., MgGTP) salts dissolved in our internal solution, yielded to comparable traces as depicted in Supplementary Figure 1.

The discharge pattern and intrinsic properties of each recorded cell were disclosed by injecting 1-s-long hyperpolarizing and depolarizing current steps. During these current injections a given neuron always remained at the same membrane potential (variable between neurons), those neurons with high spontaneous activity were hyperpolarized until they became silent, all recordings were performed in the current-clamp mode. As we did not correct for liquid junction potentials, our membrane voltage values may change in a range of 10–15 mV. The electrophysiological variables measured for each neuron were: **resting membrane potential (Vm)**, the trans-membrane voltage measured immediately after obtaining stable whole-cell configuration; **action potential** (**AP) threshold**, measured as the most negative voltage value reached by the cell prior to the beginning of the inflection for the all-or-none AP, for each cell at least five depolarizing current injections were done to test this variable, always maintaining Vm; **sag potential**, measured as the difference between the peak voltage displacement and the steady-state voltage evoked by a 1-s hyperpolarizing current injection that drove the Vm beyond −80 mV and up to −100 mV; differences ≥ 2 mV were considered as sag potentials, all measurements were obtained from at least five current injections; **membrane time constant (τm)**, calculated by fitting an exponential curve to the decay phase of a depolarizing sub-threshold stimulus; **AP frequency**, measured as the number of APs during a 1-s suprathreshold depolarizing stimulus. First, we defined the threshold depolarizing stimulus as the minimal current able to reliably evoke at least one AP, and then we applied twice that current to evoke the suprathreshold spike train used for the quantifications; **rheobase**, which is the current required to elicit at least one AP; **spike frequency adaptation**, measured as *t2/t1*, where *t2* is the time between the peaks of the last two APs of the suprathreshold spike train, and *t1* is the period between the peaks of the first two APs of the suprathreshold spike train, values >1 indicate accommodation, values <1 indicate acceleration, whereas values = 1 indicate steady-state firing; **spike-width**, measured as the width (ms) of the first AP of the suprathreshold spike train, at 50% of its maximal amplitude; **input resistance**, measured in response to an hyperpolarizing stimulus.

For the neurons that exhibited voltage-dependent bursts of APs, we measured (60 measurements for each) **inter-burst interval**, defined here as the time between the peak of the last AP of a burst and the peak of the first AP of the next burst; **burst duration**, defined as the time between the first and the last AP in a single burst, and **bursting frequency**, defined as the number of AP in a burst per unit of time (seconds), before and after incubation with CNQX and D-APV (20 μM each for all experiments; Sigma, St. Louis MO). We searched for statistical significance within cells and also compared the mean values of each parameter measured in five neurons before and after drug application using paired *t*-tests.

To assess connectivity between the MOB and AOB and within the AOB, field stimulation was applied to the dorsal MOB, situated immediately anterior to the olfactory limbus (Larriva-Sahd, 2012b) and to the pAOB when recording in aAOB, whereas dorsal MOB or pAOB field stimulation was applied when recording in aAOB. The field electrical stimuli were delivered with a concentric bipolar electrode, which had an inter-polar distance of 50 μm at the tip (Peña et al., 2002, 2010; Zavala-Tecuapetla et al., 2014). Brief square current pulses (100 μs, 0.05 Hz) were applied, while the stimulus intensity was varied according to the response elicited. We utilized a sampling frequency of 1 KHz and did corroborative experiments sampling at 10 KHz. Recordings were performed with a HS-2 headstage (Molecular devices) which has a gain of 0.01 MU. Signals were recorded on a computer using an analog-to-digital converter (BNC-2110, National Instruments) and stored on a personal computer using a custom-made program (Lemus-Aguilar et al., 2006) and an acquisition system from National Instruments (Austin, TX).

### Histochemistry

To identify recorded neurons (1 per slice in most cases), 1% biocytin was included in the pipette solution (Zavala-Tecuapetla et al., 2014). Recordings lasted between 30 and 120 min; during that time biocytin diffused into distal processes (Zavala-Tecuapetla et al., 2014). Once the recording was finalized, the pipette was gently removed to avoid damage of the neuron's somata, and slices were fixed for at least 2 days in 0.1 M phosphate-buffered saline (pH 7.4, 4°C) with 4% paraformaldehyde and 1% picric acid (Zavala-Tecuapetla et al., 2014). Next, slices were thoroughly washed three times in 1x KPBS to remove excessive fixative, endogenous peroxidase activity was blocked by incubating slices for 60 min in 3% H_2_O_2_ diluted in PBS, slices were then washed again three times with PBS and stained overnight in TBS containing Triton X-100 and avidin-biotin-peroxidase complex (1:100; Vector Laboratories, Burlingham, CA) at room temperature. The next day, after washing three times with KPBS, slices were incubated in 3,3′-diaminobenzidine (DAB) tetrahydrochloride (0.05%) and H_2_O_2_ (0.003%) in TBS (Zavala-Tecuapetla et al., 2014). In some cases, slices were further cleared in order to fully visualize distal axons. Briefly, slices were dehydrated in graded methyl-alcohols followed by a 30-min incubation in absolute methanol and benzyl alcohol: benzyl-benzoate (BABB) solution (1:2; MP Biomedicals, Aurora, Ohio). Finally, the slices were mounted on slides to visualize the reaction product of the bound horseradish peroxidase using the light microscope.

### Data analysis

Neuronal somata, dendrites, axons, and collaterals were reproduced with a camera lucida adapted to a Zeiss Axioplan 2 microscope, utilizing 10x, 40x, and 100x objectives (NA = 0.3, 1.0, and 1.4, respectively) or digitized and measured with a personal computer aided by Kontron 400 software. Measurements included somata largest and transverse axes, somata area (expressed in μm and μm^2^, respectively) and number of glomerular dendrites. These measurements were made in 44 successfully recovered and visualized neurons; data are expressed as means ± SEM unless stated otherwise.

## Results

### Electrophysiological properties of AOB large principal cells

In the present study we report whole-cell patch-clamp recordings of 92 neurons of the anterior and posterior regions of the AOB made with the primary goal of determining connectivity principles within the AOB and between the latter and the MOB. When recordings were performed on the aAOB (*n* = 72), field electrical stimulation was applied in the dorsal-posterior region of the MOB and in the pAOB, whereas recordings from the pAOB (*n* = 20) were accompanied by field electric stimulation of the aAOB and the MOB. Twenty-eight neurons (30%) elicited an antidromic and/or an orthodromic response following stimulation of the regions mentioned (Figures 5–7; see below).

**Figure 1:**
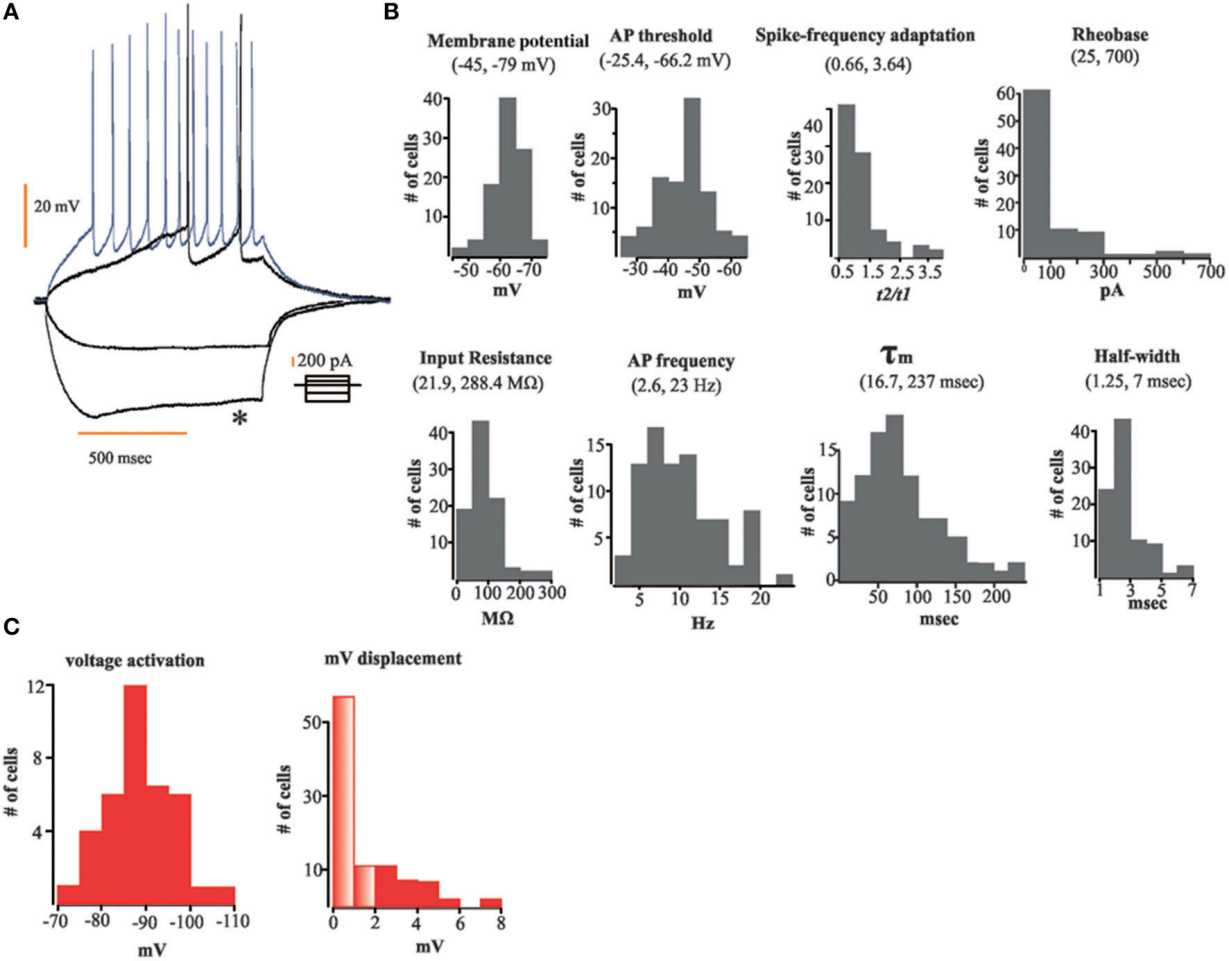
**Data distribution (histograms) of the electrophysiological variables measured**. **(A)** Representative traces of our current injection protocol; depolarizing and hyperpolarizing current steps were applied to each neuron to disclose their electric properties; note sag potential (asterisk). **(B)** Histograms of electrophysiological variables measured; from top to bottom and left to right: membrane potential, action potential (AP), threshold, spike-frequency adaptation, Rheobase, input resistance, AP frequency, τm, and half-width. **(C)** Frequency histograms of voltage activation (left) and voltage displacement (right) of neurons showing sag potential (*n* = 35); transparent bars designate number of cells without a sag potential (differences ≤ 2 mV between peak and steady-state membrane voltage upon hyperpolarizing current injection).

For the entire population of recorded neurons we measured specific electrophysiological variables (see Methods Section and Figure 1). For each neuron recorded, we first applied a series of depolarizing and hyperpolarizing current injections (Figure 1A) in order to obtain the following population parameters (mean ± SEM): **Vm** = 63.5 ± 0.51 mV; **τm** = 85.4 ± 5.2 ms; **Rheobase** = 129.7 ± 15 pA; **AP threshold** = 45.45 ± 0.86 mV; **spike frequency adaptation** = 1.23 ± 0.07; **half-width** = 2.72 ± 0.12 ms; **Input resistance** = 88.43 ± 5 MΩ; **AP frequency** = 10.21 ± 0.51 Hz. Figure 1B shows frequency histograms of the physiological variables measured. We found that from the entire population of recorded neurons only 40% (*n* = 37) of them displayed a sag potential (*I*_*h*_ current; Figure 1C). Twenty-eight were from the aAOB and nine from the pAOB. This feature has been shown to be an important network affiliation signature in the MOB (Angelo et al., 2012). We have confirmed some intrinsic electrophysiological characteristics of AOB LPCs, which respond to threshold-surpassing stimuli with few APs and have a significant delay in their AP onset (see Figure 1B), these properties differ from those seen in mitral cells of the MOB (Zibman et al., 2011). We also find neurons that exhibit accommodating, non-accommodating, and steady-state firing responses as reported previously (see Figures 1A,B); (Zibman et al., 2011).

**Figure 2:**
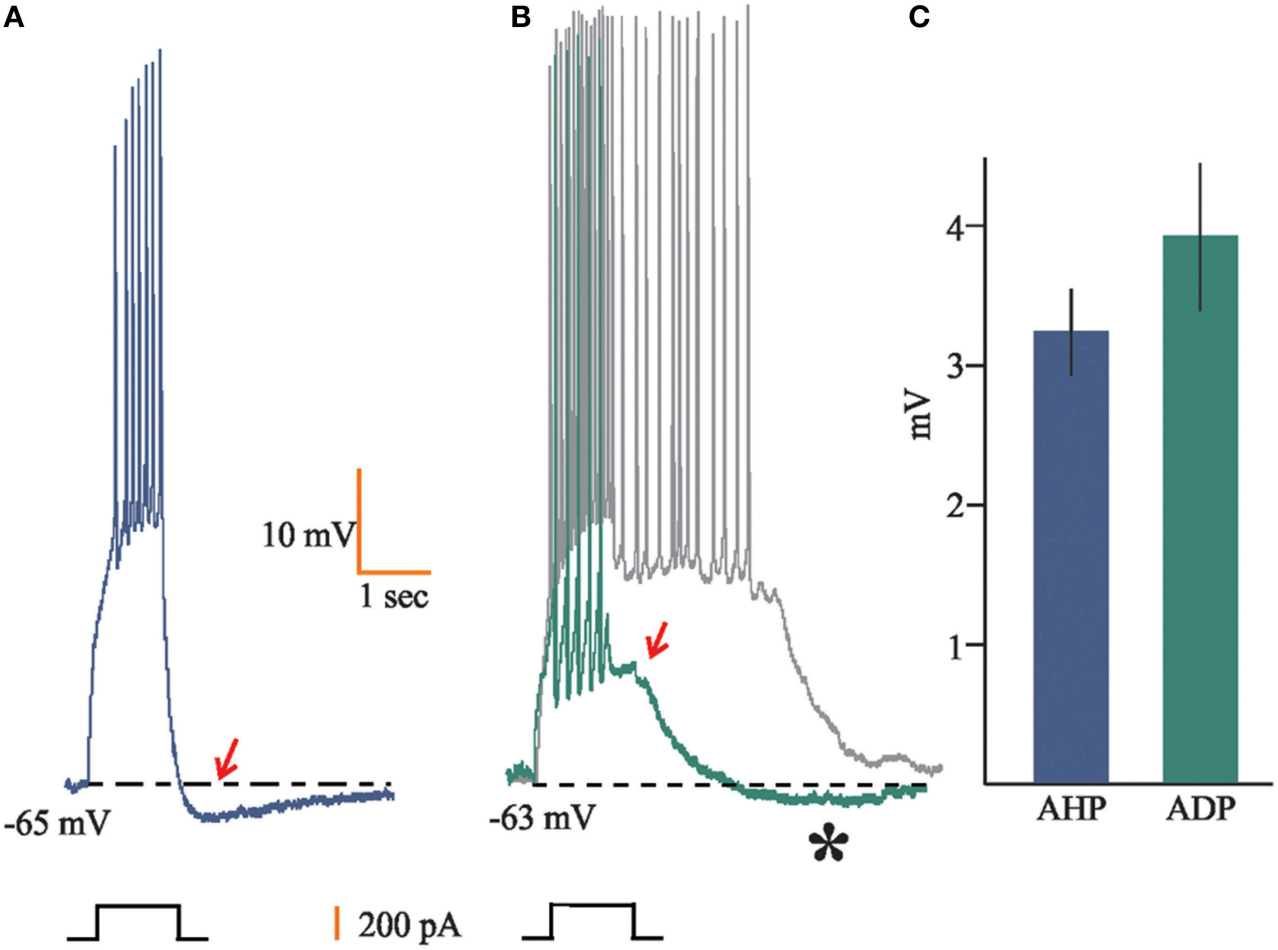
**Afterpotentials in accessory olfactory bulb (AOB) neurons**. **(A)** 42% of the neurons recorded (*n* = 39) displayed after hyperpolarization (AHP) (arrow) upon depolarizing current steps, **(B)** whereas seven and nine cells (17% in total) showed after depolarization (ADP) (arrow) or persistent firing (gray trace), respectively. Some cells (*n* = 6) also displayed slowly activating AHP following the ADP (asterisk). **(C)** Mean ± SEM of voltage displacements for AHP and ADP population values.

Additionally, we found that 42% of the recorded neurons (*n* = 39) showed a characteristic after-hyperpolarization (AHP; 3.2 ± 0.31 mV) following the train of APs evoked by the suprathreshold depolarizing current steps (Figures 2A,C). Conversely, 17% of the neurons recorded (*n* = 16) displayed after-depolarizations (ADP), for which we measured the amplitude of the voltage displacements (3.94 ± 0.52 Mv; Figure 2C). In nine of these cells such ADPs reached a plateau that elicited spiking activity for 1–10 s (Figure 2B). Only six cells displayed both AHP and ADP, with the AHP emerging just at the end of the ADP (Figure 2B).

### Large principal cells of the AOB discharge voltage-dependent rhythmic bursts of action potentials

**Figure 3:**
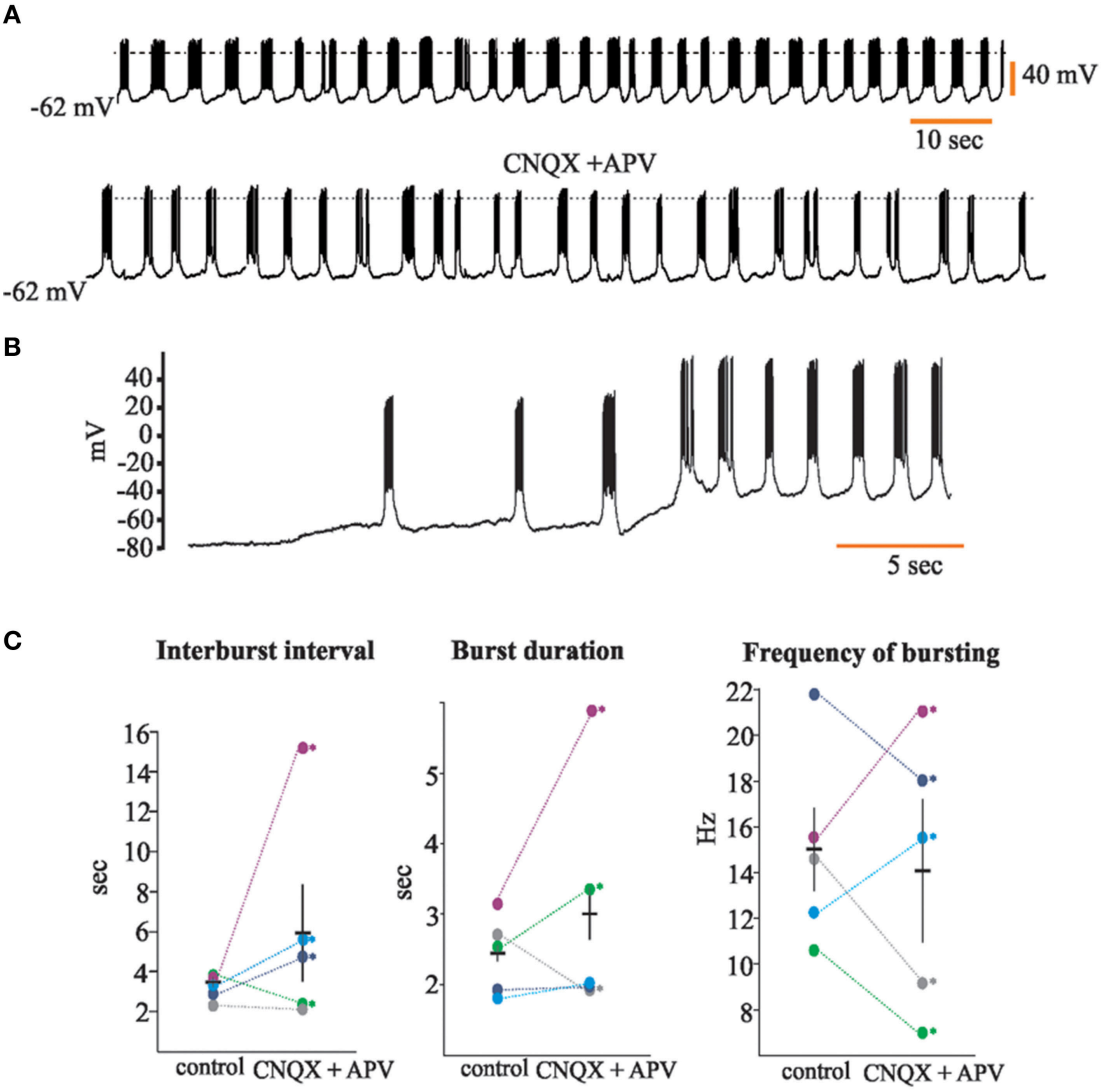
**Accessory olfactory bulb (AOB) neurons display voltage-dependent bursts of action potentials**. **(A)** Trace of spontaneous activity from a representative “rhythmic” neuron of the AOB (top); when cells are incubated with glutamatergic antagonists CNQX and APV (20 μM), rhythmic bursting persists (bottom); dotted line is 0 mV **(B)** Rhythmic firing of AOB neurons is voltage-dependent. **(C)** Dot plots showing analysis for inter-burst interval (left), burst duration (center), and bursting frequency (right). Each color represents a single neuron (*n* = 5). Statistical differences in control vs. CNQX + APV were only found within single neurons (asterisks).

We describe and confirm here the presence of neurons with intrinsic voltage-dependent rhythmic discharge patterns (Gorin, 2014; Gorin and Spehr, 2014; Figure 3) in the rat's AOB. Of the total of 92 recorded neurons, 22 (24%) of them displayed rhythmic bursts of voltage-dependent APs. We recorded 19 neurons with such characteristics in the aAOB and three in the pAOB (26 and 15%, respectively; Figure 3A). For some of these neurons (*n* = 5) we measured the inter-burst interval (3.21 ± 0.27 s), the duration of the burst (1.41 ± 0.24 s in control) and the bursting frequency (15.02 ± 1.91 Hz; Figure 3C), before and after bath-applying the glutamatergic inhibitors CNQX and APV (20 μM; Zavala-Tecuapetla et al., 2008, 2014). After blocking fast glutamatergic transmission, the rhythmic burst firing persisted, which led us to conclude that these voltage dependent bursts were generated endogenously (Peña et al., 2004; Zavala-Tecuapetla et al., 2008, 2014).

**Figure 4:**
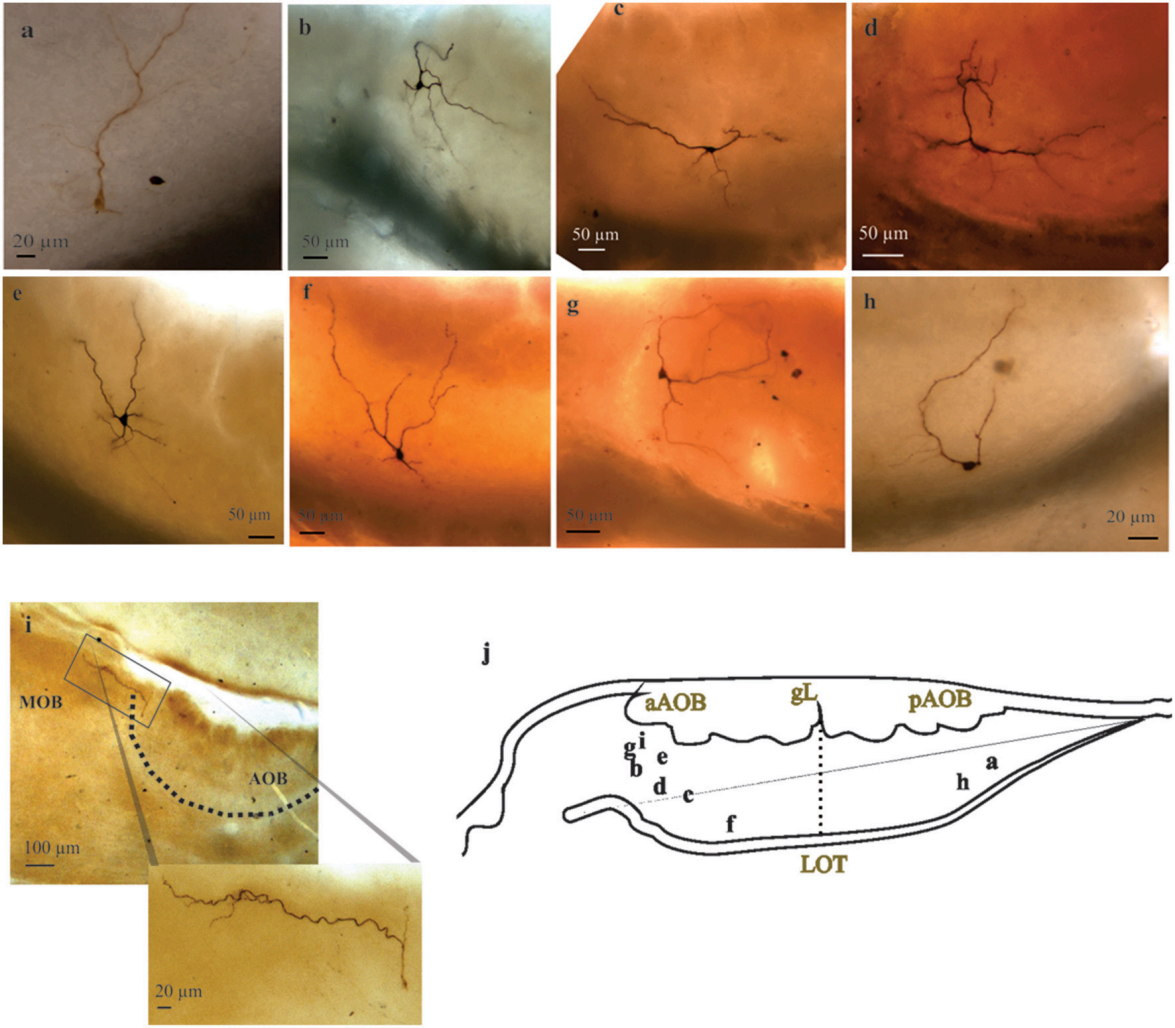
**Large principal cells of anterior and posterior halves of the accessory olfactory bulb (AOB)**. **(A–I)** Representative pictures of recorded AOB-large principal cells (LPCs). Note the variable morphology of this population of principal cells and their distinct degrees of glomerular innervation. Neuron in ***i*** is a “rhythmic” neuron, note its far reaching dendrite leaving the AOB. **(J)** Camera lucida drawing showing the approximate position of the neuron's somata.

**Figure 5:**
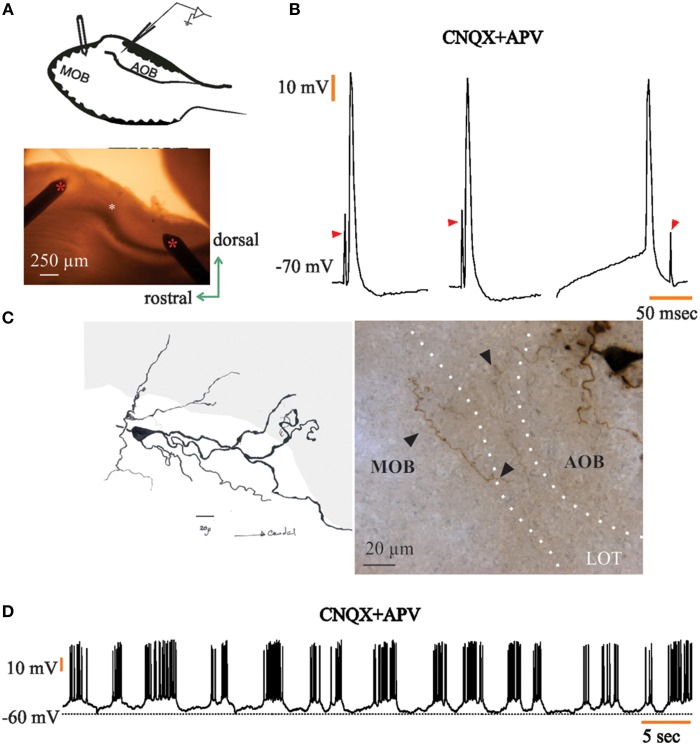
**Large principal cells of the anterior half of the accessory olfactory bulb (aAOB) send axon collaterals to the dorsal main olfactory bulb (MOB). (A)** Schematic of the recording setup (up) and picture taken the day the neuron in ***c*** was recorded (red asterisks, stimulating electrodes; white asterisk, tip of the recording electrode). **(B)** Antidromic action potentials elicited by dorsal-MOB stimulation (left); the evoked antidromic responses persist in the presence of 20 μM of CNQX and APV (center); collision tests annihilated the evoked response (right); arrowheads indicate stimulating artifact. **(C)** Drawing (left) and photomontage (right) of the LPC recorded; note that the axon (arrowheads) that has been omitted in the drawing leaves aAOB territory and heads toward the MOB. Orientation is the same as in ***a***. **(D)** The neuron recorded fires rhythmic bursts of action potentials in the presence of CNQX and APV (20 μM).

All “rhythmic” neurons that were successfully recovered for morphological analysis (*n* = 8) exhibited morphological features corresponding to LPCs (Figures 4B–D), which consist of the presence of one or more glomerular dendrites and an axon entering the lateral olfactory tract (LOT). Moreover, “rhythmic” LPCs often had elaborate dendritic arborizations innervating more than one glomerulus (Figure 4D) and, one of them seemed to be tributary of a glomerular complex far beyond aAOB confines (Figure 4I). It is also noteworthy that half (3/6) of the AOB neurons that elicited antidromic APs (aAPs) upon MOB electric stimulation exhibited oscillatory discharge patterns spontaneously (see below, Figures 3, 5).

### Cytology

The majority of visualized neurons (*n* = 41; Figure 4) corresponded to LPCs of either anterior or posterior AOB halves and they were classified according to their anatomical characteristics, namely, their size (Takami and Graziadei, 1991), morphology (i.e., glomerular dendrites), and axonal arborization, which frequently incorporates and proceeds in the LOT (Mohedano-Moriano et al., 2007; Larriva-Sahd, 2008). We measured somata length (20.41 ± 0.87 μm), width (12.47 ± 0.64 μm), area (204.44 ± 18.44 μm^2^), and number of glomerular dendrites (3.1 ± 0.23) for the neurons that were successfully recovered for morphological characterization (*n* = 44).

As described elsewhere (Larriva-Sahd, 2008), LPCs visualized here were scattered along the external cellular layer (ECL) of the AOB providing glomerular dendrites to more than one glomerulus (Figure 4), and, occasionally, to up to six glomeruli. These are important differences between mitral and LPCs (Hayar et al., 2004a; Larriva-Sahd, 2008; Zibman et al., 2011).

### aAOB large principal cells send axon collaterals to the dorsal MOB

For the neurons recorded in the aAOB (*n* = 72), we found that a proportion of them (*n* = 6, 8%) responded to MOB electrical stimulation with aAPs (Figure 5B, left). aAPs were evoked in an all-or-none fashion and had a very short latency (1.97 ± 0.02 ms). These responses persisted in spite of the presence of the glutamate inhibitors CNQX and APV (20 μM) (Figure 5B, center), disclosing the non-synaptic nature of the evoked APs; furthermore, collision tests prevented aAP generation after inducing a somatic AP (Figure 5B, right), which led us to the conclusion that LPCs in the aAOB must send axons to the dorsal region of the MOB.

Indeed, the *post-hoc* visualization of the neurons with antidromic responses revealed that they emit axon collaterals directed toward the MOB (e.g., the stimulation site; Figure 5C). The somato-dendritic features of the neurons that responded with aAPs resembled those of LPCs of the AOB (Takami and Graziadei, 1991; Larriva-Sahd, 2008); moreover, the presence of glomerular dendrites and their distinctive axon entering the LOT served as an unequivocal determinant of cell identity; hence, we conclude that there is a sub-population of LPCs of the aAOB that sends axon collaterals to the the dorsal-posterior region of the MOB. Interestingly, three of the six neurons that were antidromically activated by applying electrical stimuli to the MOB, displayed a voltage-dependent burst discharge pattern that was maintained in the presence of glutamatergic inhibitors (Figure 5D).

### Anterior and posterior halves of the AOB are reciprocally connected

**Figure 6:**
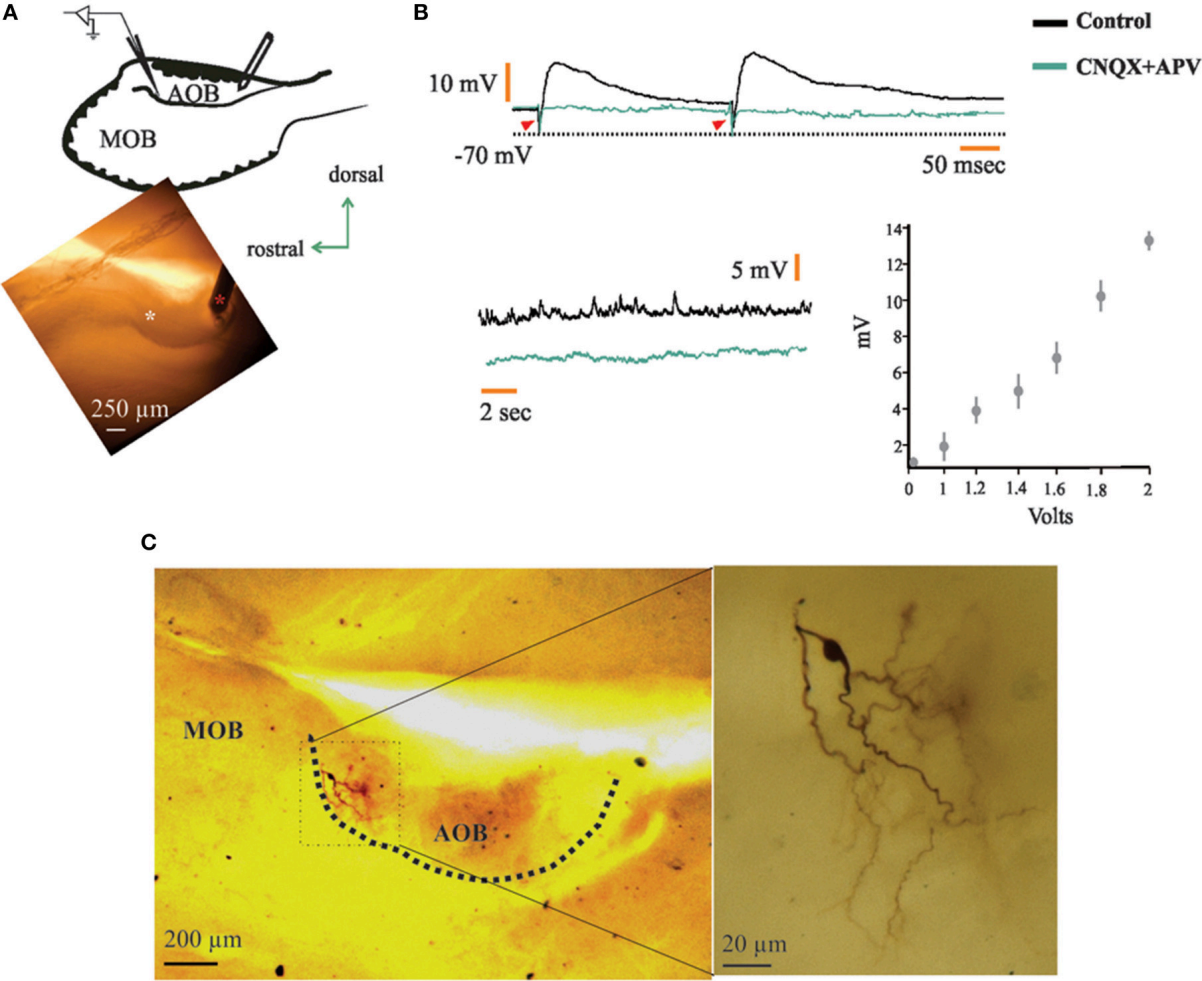
**Neurons in the anterior half of the accessory olfactory bulb (aAOB) receive synaptic input from posterior half (pAOB) neurons**. **(A)** Schematic of the recording setup (left) and picture taken the day the neuron in ***c*** was recorded (red asterisk, stimulating electrode; white asterisk, tip of recording electrode). **(B)** Top: upon pAOB paired-pulse stimulation, neurons (*n* = 6) responded with excitatory postsynaptic potentials (EPSPs) that were completely abolished in the presence of CNQX and APV (20 μM; arrowheads indicate stimulating artifact). Bottom: spontaneous EPSPs before and after CNQX and APV incubation (left) and plot showing EPSP response amplitude vs. stimulus intensity (right; *n* = 6). **(C)** Picture of the neuron at low (left) and high (right) magnifications, note the very anterior position of the cell recorded (top left), which has a morphology resembling that of a short-axon neuron.

**Figure 7:**
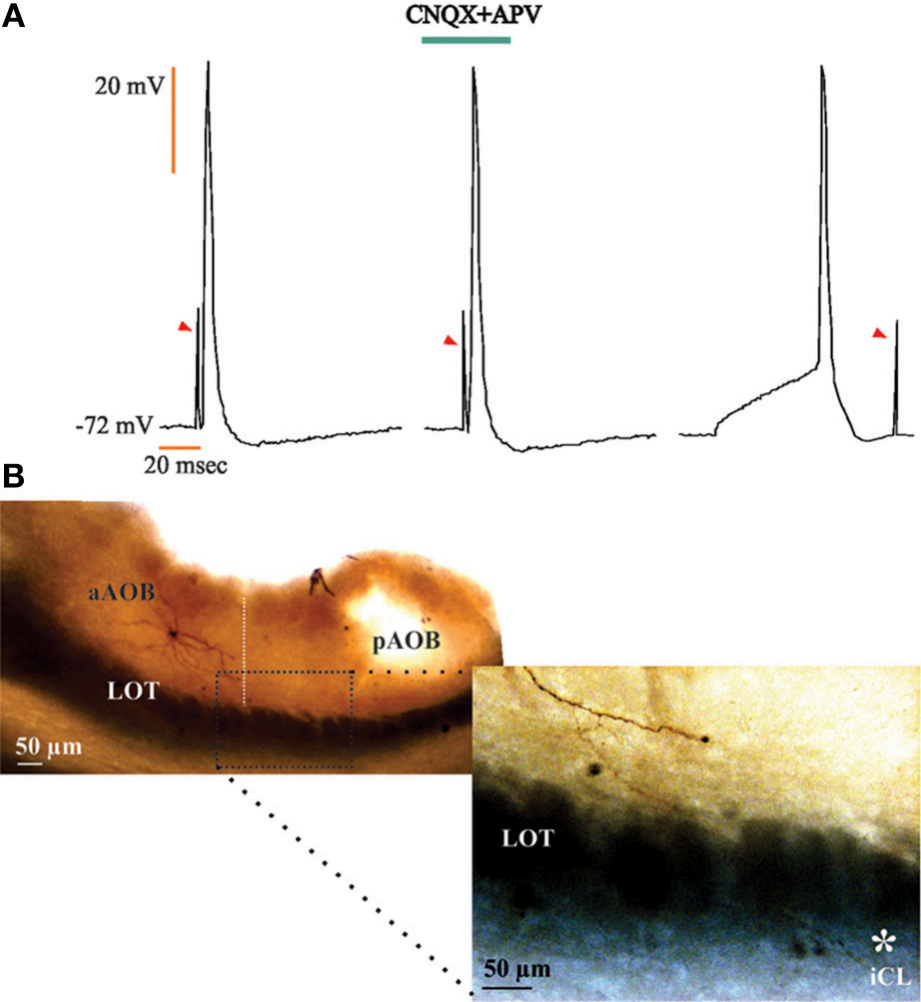
**Large principal cells in the anterior half of the accessory olfactory bulb (aAOB) send axons to the homonymous cells in the posterior half (pAOB)**. **(A)** Action potentials evoked by opposite half AOB field stimulation (left); these responses persist in the presence of CNQX and APV (20 μM; center) and were always collided (right). Arrowheads: stimulation artifact. **(B)** Low power photograph of anterior and posterior AOB halves (upper left); an aAOB neuron sends a collateral fiber^*^ that resolves in the opposite internal cellular layer (ICL; lower right).

When we applied electrical stimuli in the pAOB while recording in the aAOB (Figure 6A) we found that neurons responded with either orthodromic excitatory post-synaptic potentials (EPSPs; *n* = 6, 7.5%; Figure 6B) or with aAPs (*n* = 8, 11%; Figure 7A, left). The half-width of the EPSPs was of 120.89 ± 18.65 (mean ± SEM) which may be due to the combined activation of NMDA and non-NMDA receptors (Forsythe and Westbrook, 1988; Trombley and Westbrook, 1990; Maccaferri and Dingledine, 2002). Five out of six neurons had latencies of 3.07 ± 0.12 (mean ± sd), failure rates of 8 ± 4%, and a shock-to-shock variability (synaptic jitter) ranging from 45 to 130 μs, which may be suggestive of monosynaptic connection (Doyle and Andresen, 2001). We further evaluated if these responses were mediated by glutamatergic transmission by bath-applying CNQX and APV (20 μM) into the recording chamber and found that indeed, EPSPs were completely abolished after the pharmacological blockade of excitatory synaptic transmission (Figure 6B). This strongly suggests that there might be a monosynaptic connection arising from pAOB neurons. Two of the six neurons that elicited EPSPs upon activation of their opposite half displayed morphological features consistent with those of interneurons, namely, a dense peri-somatic axonal arborization that avoids the LOT and the lack of glomerular dendrites (Figure 6C); whereas the remaining neurons exhibited LPC morphology.

For the neurons recorded in the aAOB that evoked aAPs due to the electrical activation of the pAOB, we found that these responses persisted in the presence of glutamatergic inhibitors and also, collision tests resulted in annihilation of the evoked aAPs (Figure 7A). Furthermore, histological inspection of these cells showed that they correspond to LPCs that display axon collaterals that cross the *línea alba* (LA; see Larriva-Sahd, 2008) and may influence pAOB neurons before incorporating into the LOT (Figure 7B).

We last performed a subset of recordings in the pAOB in order to determine if the synaptic activation seen in aAOB when stimulating the pAOB is a reciprocal event. Thus, we recorded from cells in the pAOB (*n* = 20) while applying electrical stimuli to the aAOB. Indeed, neurons in the pAOB responded to the electrical stimulus with either aAP or with orthodromic EPSPs (*n* = 7 and 1, respectively). As in the previous experiments, the aAPs were prevented in collision tests, and they persisted in the presence of glutamatergic inhibitors, whereas the EPSPs were abolished by CNQX and APV bath application. Altogether these results lead us to conclude that both AOB halves are linked by glutamatergic synapses mediated by the LPCs of both halves, whose common target may be other LPCs or putative short-axon neurons.

## Discussion

Here, we investigated the inner circuitry of the AOB and its interaction with the MOB. Previous structural and physiological studies suggested that, in sharp contrast with the MOB, the AOB is parceled along the antero-posterior axis into two distinct halves. Both AOB halves are interconnected by at least two sets of distinct processes, namely, LPC axons and its dendrites (Larriva-Sahd, 2008).

LPC axons zigzag in the rostro-caudal direction providing defined sets of collaterals to both halves; accessory dendrites, on the other hand, pierce the LA to resolve in the neuropil of the adjacent AOB half. Since axon collaterals issued by the LPC axon appear to terminate on interneurons in the opposite AOB half, as suggested by the Golgi technique, a basic circuitry between the two AOB halves was furnished (Larriva-Sahd, 2008). With this background, *in vitro* whole-cell patch-clamp recordings of adult rat olfactory bulb were utilized in the present study to depict possible synaptic interactions between the aAOB and pAOB. Recordings confirmed that principal cells of both halves project collaterals piercing the LA to resolve in the opposite AOB half.

The presence and unique intrinsic properties (Gorin, 2014; Gorin and Spehr, 2014) of AOB “rhythmic” LPCs (Figure 3) suggests that they may play a significant role in information processing within the AOB. Furthermore, a subset of the oscillatory aAOB LPCs sends axon collaterals to the dorsal part of the MOB, which suggested us the possibility that there might be a direct, functional synaptic link between the aAOB and dorsal MOB. Santiago Ramón y Cajal defined that granule cells in the homonymous layer represent the converging site of extrinsic modulatory influences on the mitral cell (Larriva-Sahd, 2012a). This, coupled with our neurophysiological evidence suggests that APs generated by LPC pheromonal recruitment may have, in turn, a granule cell-mediated influence on the MOB-mitral cell (see Martínez-García et al., 1991 and Pressler and Strowbridge, 2006).

### Both AOB halves possess a sub-population of “Rhythmic” large principal cells

The confirmation (Gorin, 2014; Gorin and Spehr, 2014) that a set of LPCs corresponds to typical “pacemaker” neurons is potentially important in the context of both AOB circuitry and its projection to the MOB; 24% of our sample corresponds to “rhythmic neurons.” This electrophysiological profile is generated endogenously in LPCs of the rat's AOB (Figure 3A), and it is voltage-dependent (Figure 3B). Pacemaker-like cells have been previously found in the MOB, where external tufted neurons (eT) fire intrinsically generating rhythmic bursts of action potentials (Hayar et al., 2004a,b; Liu and Shipley, 2008), and recently, Golgi cells of the MOB granule layer have also been reported to fire state-dependent rhythmic discharges (Pressler et al., 2013).

LPCs of the AOB oscillate at much higher frequencies (15.6 ± 1.91 Hz) than their counterparts in the MOB (see Figure 3), as it is reported that Golgi and eT cells fire near the θ frequency (Hayar et al., 2004a; Pressler et al., 2013), which suggests that these rhythmic discharges parallel the sniffing cycles. In contrast, access of stimuli to the VNO is aided by mechanical (Meredith and O'Connell, 1979) and behavioral (Mann, 1961) processes. Thus, the dissimilar dynamics of central processing reflected by the bursting frequency may also underlie the functional differences observed between the two systems.

Another important difference between AOB and MOB “rhythmic” cells is their dendritic arborization. In fact, almost all eT cells have only one glomerular dendrite that extensively ramifies within a single tributary glomerulus (Hayar et al., 2004a) which contrasts to the numerous glomerular dendrites we have seen in our LPCs (3.1 ± 0.23). Moreover, glomerular dendrites often were committed to at least two glomeruli and ramified less profusely within the glomerular domain (see Figure 4; see Larriva-Sahd, 2008).

The functional significance of the rhythmic activity observed in the AOB remains to be determined. However, due to the fact that each glomerulus may receive sensory input from more than one receptor type (Belluscio et al., 1999; Wagner et al., 2006) and that each LPC innervates more than one glomerulus (Larriva-Sahd, 2008), it is possible that these cells may serve as network synchronizers (Hayar et al., 2004b; Peña et al., 2004; Ramírez et al., 2004).

Whatever the post-synaptic effect(s) of the episodic bursting of LPCs on their eventual targets might be, they are committed to pheromone detection (Leinders-Zufall et al., 2000; Boschat et al., 2002; Del Punta et al., 2002; Luo et al., 2003) and some LPCs exhibiting “rhythmic” discharges project to the MOB. Hence, it is plausible that certain pheromones may have a modulatory influence on the latter via the AOB-MOB interaction documented here. Although in our sample (*n* = 5) of “rhythmic” cells treated with glutamatergic inhibitors we did not find statistical differences in bursting properties before and after bath-applying CNQX and APV (Figure 3C), it is clear that glutamatergic modulation may affect certain parameters of their rhythmic bursting differentially (see Figure 3C). Moreover, we recorded two neurons in which both synaptic excitation and inhibition were blocked and the rhythmic activity was still observed (data not shown).

### Electrophysiological properties of large principal cells in the AOB

As already mentioned, the majority of neurons recorded and successfully visualized corresponded to LPCs and, although not our major goal in this research, we defined some of their electrophysiological characteristics that might be relevant. For instance, we found that principal cells in the AOB in either half may have persistent firing activity upon cessation of stimuli. Neurons exhibiting such properties, have been shown both *in vitro* (Shpak et al., 2012) and *in vivo* (Luo et al., 2003; Figure 2B). It has been suggested that these intrinsic properties may be associated with social context decoding (Shpak et al., 2012). Furthermore, some of these cells fire persistently and some others display ADPs that do not develop into persistent firing. Both characteristics result from distinct biophysical mechanisms (Shpak et al., 2015) and may also be modulated by basal forebrain cholinergic inputs (Smith and Araneda, 2010; Shpak et al., 2015). Because of both the basic physiological properties of these neurons and the extrinsic modulatory influence they receive, it is conceivable that they are involved in decoding complex sensory cues.

### Axonal link between the accessory- and main-olfactory bulbs

The existence of the axonal projection described here from the aAOB to the dorsal-posterior MOB (Figure 5) may represent one anatomical substrate accounting for the functional cross-talk observed between the main- and accessory-olfactory systems (Xu et al., 2005; see Baum and Larriva-Sahd, 2014). Consistent with this interpretation is that the dorsal MOB, a region receiving axonal collaterals from the AOB (Figure 5), has been implicated in the expression of social behaviors in mice (Matsuo et al., 2015).

Potentially relevant is the observation that at least a set of LPCs displaying “rhythmic” activity projects from the aAOB to the dorsal MOB. Hence, these cells may imprint a pre-synaptic, synchronizing activity upon the MOB. Moreover, the fact that at least one of the recorded rhythmic neurons issued a long dendrite encompassing what seems to be a modified MOB glomerular complex (Shinoda et al., 1989; see Figure 4I) suggests convergence of olfactory and vomeronasal afferents into a single LPC. If there is a reciprocal (i.e., between the MOB to AOB) projection remains to be determined; however, electrolytic damage of the dorsal MOB resulted in orthograde degeneration in the AOB neuropil (Larriva-Sahd, 2008), suggesting a mutual connectivity between them. Further, reciprocal connections between the AOB and the MOB have been reported in the reptile *P. hispanica* (Martínez-García et al., 1991). Thus far, we assume that the projections of the aAOB to the MOB are a numerically small contingent of fibers, although a systematic search for MOB projections other than from its dorsal-posterior region is required.

### The accessory olfactory bulb halves are reciprocally connected by large principal cells

The initial observation in Golgi-impregnated specimens (Larriva-Sahd, 2008) regarding axon distribution and collateralization at either side of the LA suggested that this distinct cell type represents the substrate for a functional interaction between the two AOB halves. This notion became a central hypothesis to be tested here. Whole-cell recordings performed at either side of the LA proved that LPCs are mutually connected. Visualization of LPCs following recordings revealed that their axon collaterals distribute in the adjacent AOB half (Figure 7). Furthermore, a set of distinct dendrites traversing the LA may also represent a structural link between the AOB halves (Larriva-Sahd, 2008). While a first choice strategy to define neuron to neuron interactions is the recording of cell pairs, this turned out to be technically inaccessible, at least in our hands. In fact, LPCs laying at either side of LA are far apart (>300 μm; Larriva-Sahd, 2008), which significantly lowers the probability of successfully recording synaptically linked cells (McGarry et al., 2010).

**Figure 8:**
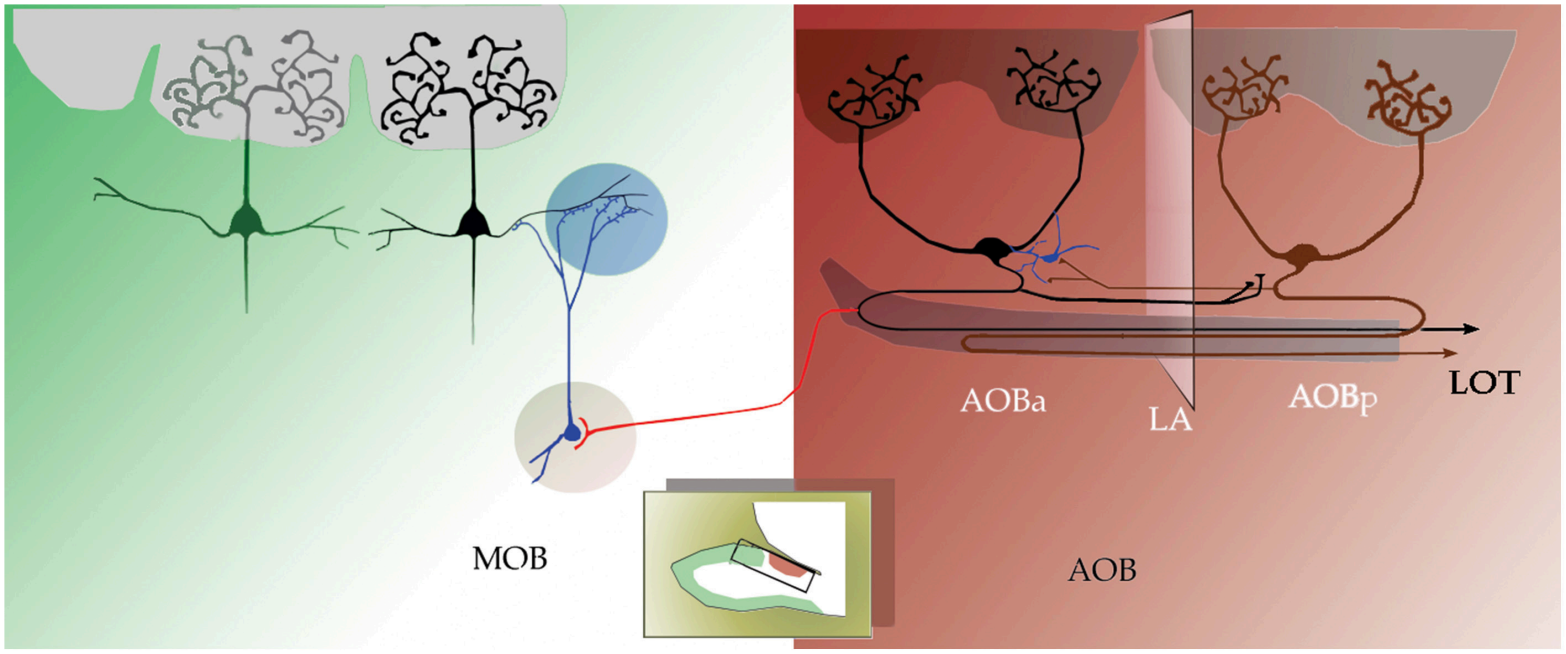
**Schematic representation of the results reported here; drawing of a sagittal view of the rat's OB**. Anterior and posterior halves of the accessory olfactory bulb (aAOB and pAOB, respectively) are interconnected by large principal cells (black, dark red) that send axons (in black and dark red) and collaterals across the *línea alba*. These axons may influence putative interneurons (blue) and this communication is reciprocal. Conversely, aAOB LPCs send collateral fibers (red) into dorsal-MOB territory.

Histological inspection of neurons that elicited EPSPs following stimulation of the opposite half suggests that some of them correspond to interneurons. Although a systematic study of neurons synaptically linked with the opposite AOB half is required, some neurons studied here exhibited dense peri-somatic axonal arborizations and absence of glomerular dendrites (Figure 6C). Thereby suggesting that short axon neurons mediate between LPCs in either AOB side (Figure 8). Hence, the structural evidence suggested earlier and confirmed here, coupled with the present physiological observations offers a normative foundation for the eventual understanding of the cellular interactions implicated in central pheromonal decoding.

Recently, an important imaging study of presynaptic calcium activity upon glomeruli, showed that AOB neurons are selectively tuned to the sex, strain, and species of urine samples (Hammen et al., 2014). Moreover, it has even been assumed that the AOB has a “modular organization” based on the defined sensory innervation of glomeruli by vomeronasal sensory neurons with similar receptive properties.

Like in the cerebral cortex, axon collaterals of principal cells recruit neighboring interneurons to define distinct functional clusters or domains (Lorente de Nó, 1949; Larriva-Sahd, 2008). The same seems to apply to LPCs that, by a set of tiny axon collaterals may recruit both, neighboring interneurons (i.e., columns or functional modules) or homonymous cells in their opposite half. Indeed, an *in vivo* study has highlighted the importance of lateral inhibition phenomena for AOB (Luo et al., 2003).

## Conclusions

There is a novel direct axonal (i.e., collateral fibers) projection from aAOB neurons into dorsal MOB territory. Second, LPCs at either side of the LA send collateral axons and terminals to their opposite side, which may establish mono-synaptic contacts with LPCs and/or putative interneurons (see Figure 8). Lastly, there is a sub-population of “rhythmic” neurons that fire voltage-dependent bursts of action potentials. These neurons reside in both halves of the AOB.

## Funding

VV is a doctoral student from Programa de Doctorado en Ciencias Biomédicas, Universidad Nacional Autónoma de México (UNAM, IN206511), and received fellowship 289638 from CONACyT.

### Conflict of interest statement

The authors declare that the research was conducted in the absence of any commercial or financial relationships that could be construed as a potential conflict of interest.

## Supplementary material

Supplementary Figure 1 **Comparable recordings of APs with MgGTP instead of LiGTP in the internal solution. (A)** Single APs obtained with LiGTP in our internal solution (black traces) and replacing the former with MgGTP (light blue); note the different time scales. **(B)** “Rhythmic” LPC of the aAOB recorded with an internal solution containing MgGTP.

## Abbreviations

aAP: antidromic action potential
AP: action potential
aAOB: anterior accessory olfactory bulb
AC: acommodating
aCSF: artificial cerebro-spinal fluid
ADP: after-depolarization
AHP: after-hyperpolarization
AOB: accessory olfactory bulb
AOS: accessory olfactory system
ECL: external cellular layer
EPSPs: excitatory post-synaptic potentials
eT: external tufted cells
FPR: formyl-peptide receptor
LA: línea alba
LOT: lateral olfactory tract
LPC: large principal cell
maCSF: modified artificial cerebro-spinal fluid
MUPs: major urinary proteins
MHC: major histocompatibility complex
MOE: main olfactory epithelium
MOS: main olfactory system
MOB: main olfactory bulb
NAC: non-acommodating
OR: olfactory receptor
pAOB: posterior accessory olfactory bulb
Vm_v_: resting membrane potential
VNO: vomeronasal organ
VR1: vomeronasal receptor 1
VR2: vomeronasal receptor 2.
