# Supplementary figures and images for: Electrophysiological Evidence for a Direct Link between the Main and Accessory Olfactory Bulbs in the Adult Rat
Source: Front Neurosci. 2016 Jan 26;9:518. doi: 10.3389/fnins.2015.00518 (PMC4726767; doi:10.3389/fnins.2015.00518)

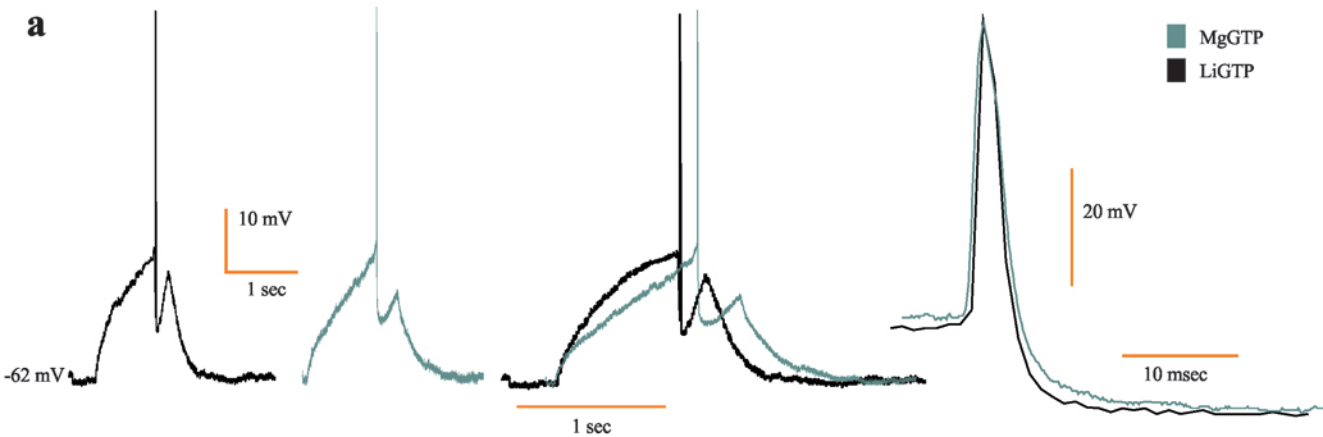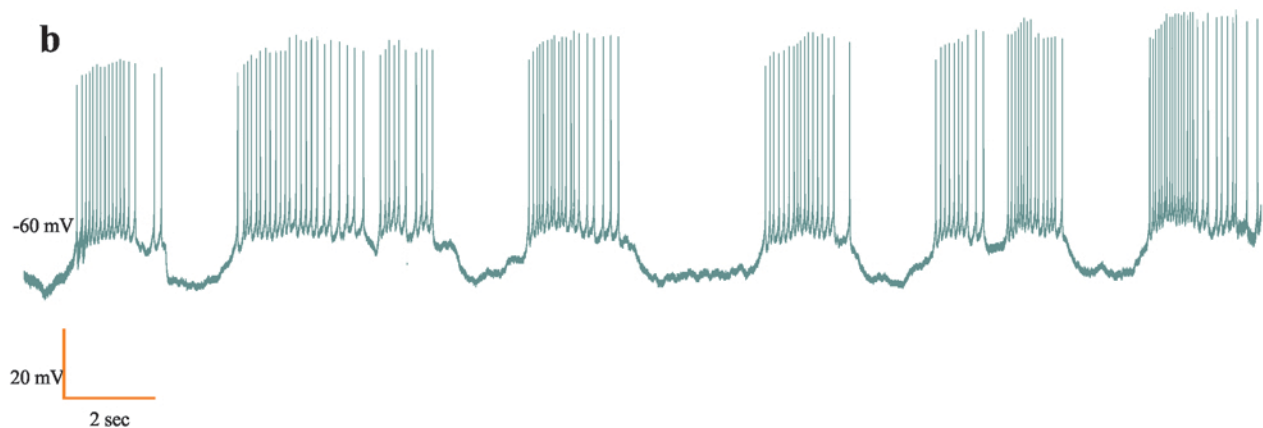

Supplement: Supplementary Figure 1 — Comparable recordings of APs with MgGTP instead of LiGTP in the internal solution. (A) Single APs obtained with LiGTP in our internal solution (black traces) and replacing the former with MgGTP (light blue); note the different time scales. (B) “Rhythmic” LPC of the aAOB recorded with an internal solution containing MgGTP. [file DataSheet1.PDF]
